# First-Line Atezolizumab Plus Bevacizumab versus Sorafenib in Hepatocellular Carcinoma: A Cost-Effectiveness Analysis

**DOI:** 10.3390/cancers13050931

**Published:** 2021-02-24

**Authors:** Chi-Leung Chiang, Sik-Kwan Chan, Shing-Fung Lee, Horace Cheuk-Wai Choi

**Affiliations:** 1 Department of Clinical Oncology, University of Hong Kong, Hong Kong, China; csk01@connect.hku.hk (S.-K.C.); hcchoi@hku.hk (H.C.-W.C.); 2 Department of Clinical Oncology, Tuen Mun Hospital, Hong Kong, China; bestmic@hotmail.com

**Keywords:** atezolizumab plus bevacizumab, hepatocellular carcinoma, cost-effective analysis

## Abstract

**Simple Summary:**

There is a growing body of literature demonstrating high cancer drug costs relative to the benefits provided to patients treated on a large scale. We examined the cost-effectiveness of atezolizumab–bevacizumab for the first-line treatment of patients with unresectable hepatocellular carcinoma, based on the results of the pivotal phase 3 trial IMbrave 150. Our model was most sensitive to the overall survival hazard ratio and body weight. We found that atezolizumab–bevacizumab was cost-effective if we assumed all patients at the end of the IMbrave 150 trial were cured of hepatocellular carcinoma. Otherwise, atezolizumab–bevacizumab was not cost-effective. We concluded that price reduction, duration of therapy capped to ≤12 months, or dosage of bevacizumab reduced to ≤10 mg/kg would favorably influence cost-effectiveness, even if long-term clinical benefits were modest. The long-term effectiveness of atezolizumab–bevacizumab is a critical factor of its cost-effectiveness. Further studies to optimize the duration and dosage of therapy are warranted.

**Abstract:**

Background: The IMbrave 150 trial revealed that atezolizumab plus bevacizumab (atezo–bev) improves survival in patients with unresectable hepatocellular carcinoma (HCC) (1 year survival rate: 67.2% vs. 54.6%). We assessed the cost-effectiveness of atezo–bev vs. sorafenib as first-line therapy in patients with unresectable HCC from the US payer perspective. Methods: Using data from the IMbrave 150, we developed a Markov model to compare the lifetime cost and efficacy of atezo–bev as first-line systemic therapy in HCC with those of sorafenib. The main outcomes were life-years, quality-adjusted life-years (QALYs), lifetime costs, and incremental cost-effectiveness ratio (ICER). Results: Atezo–bev demonstrated a gain of 0.44 QALYs, with an additional cost of USD 79,074. The ICER of atezo–bev was USD 179,729 per QALY when compared with sorafenib. The model was most sensitive to the overall survival hazard ratio and body weight. If we assumed that all patients at the end of the IMbrave 150 trial were cured of HCC, atezo–bev was cost-effective (ICER USD 53,854 per QALY). However, if all patients followed the Surveillance, Epidemiology, and End Results data, the ICER of atezo–bev was USD 385,857 per QALY. Reducing the price of atezo–bev by 20% and 29% would satisfy the USD 150,000/QALY and 100,000/QALY willingness-to-pay threshold. Moreover, capping the duration of therapy to ≤12 months or reducing the dosage of bev to ≤10 mg/kg would render atezo–bev cost-effective. Conclusions: The long-term effectiveness of atezo–bev is a critical but uncertain determinant of its cost-effectiveness. Price reduction would favorably influence cost-effectiveness, even if long-term clinical outcomes were modest. Further studies to optimize the duration and dosage of therapy are warranted.

## 1. Introduction

Hepatocellular carcinoma (HCC) is a common type of liver cancer and a leading cause of cancer deaths worldwide [1]. For over a decade, sorafenib, an anti-angiogenic multi-kinase inhibitor, was the only systemic agent available with a survival benefit in advanced HCC [2]. Recently, another multi-kinase inhibitor, lenvatinib, was approved based on its non-inferiority to sorafenib [3]. Checkpoint inhibitors have shown promising activity as second-line treatment of HCC in phase I/II studies [4,5,6]; however, a phase III trial has revealed that single-agent anti-programmed death 1 (PD-1) therapy failed to significantly improve survival as first-line therapy [7].

Currently, several immunotherapy combinations targeting programmed death-ligand 1 (PD-L1) and PD-1 pathways are being evaluated in advanced HCC [8]. Atezolizumab selectively targets PD-L1 to prevent interactions with PD-1 and B7-1 receptors, thus reversing T-cell suppression [9]. Bevacizumab is a monoclonal antibody that targets vascular endothelial growth factor (VEGF), inhibiting angiogenesis and tumor growth [10,11]. Anti-VEGF therapy also enhances anti- PD-1/PD-L1 activity by reducing VEGF-mediated immunosuppression and promoting T-cell infiltration in tumors [12,13].

The promising activity of atezolizumab plus bevacizumab (atezo–bev) was first reported in a phase 1b trial, with an objective response rate of 36% and a median progression-free survival (PFS) of 7 months [14]. The IMbrave 150 study is a global, multi-center, randomized phase III trial evaluating the efficacy and safety of atezo–bev when compared with sorafenib in unresectable HCC patients who received no prior systemic therapy. The trial demonstrated a significant improvement in overall survival (OS) (hazard ratio (HR), 0.58; 95% CI, 0.42–0.79) and PFS (HR, 0.59; 95% CI, 0.47–0.76) in patients treated with atezo–bev [15], leading to the approval of this combination by the FDA. However, due to the high cost of research and development, the legislation preventing Medicare from being able to negotiate the drug prices, and a lack of value-based pricing system, like many new cancer drugs, the atezo–bev combination is expensive. Therefore [16], it is pressing to define its value for policymakers, payers, patients, and clinicians, particularly from the global perspective given the high incidence of HCC in economically disadvantaged regions worldwide [17,18].

The therapeutic options of advanced HCC patients have been rapidly expanded in recent years; however, most approved therapies provided low economic value [19,20,21,22]. We aimed to evaluate the cost-effectiveness of atezo–bev vs. sorafenib as first-line systemic therapy in patients with unresectable HCC, from the US payer perspective. As with other novel therapies, there are considerable uncertainties about long-term survival outcomes, which in our case is an immunotherapy that may potentially cure a proportion of HCC patients. In order to explore such an uncertainty, our study estimated long-term survival rates across a range of three scenarios. Our analysis provides a robust framework for a value-based evaluation of atezo–bev when mature survival data are available in the near future.

## 2. Methods

### 2.1. Modeling without Long-Term Outcome Data

The IMbrave 150 study reported survival over 17 months based on a median follow-up duration of 8.6 months [15]. Previous studies have suggested that immunotherapy may cure a proportion of patients, and assumptions of long-term outcomes may significantly impact the cost-effectiveness analysis [23,24]. There may have been a risk of inaccurate assessment and uncertainties if we extrapolated long-term outcomes from short-term data based on a standard parametric model [25]. We addressed this issue by evaluating three scenarios covering a wide range of longer-term survival outcomes, similar to previous studies [20,21,22]. In our base case, long-term outcomes were extrapolated from short-term data of the IMbrave 150 study (i.e., 3-year overall survival rate of 37.7%). We assumed that overall survival after 17 months would follow the estimates of the US population with advanced HCC, obtained from the Surveillance, Epidemiology, and End Results (SEER) database (i.e., 3-year survival rate of 27.8%) in the pessimistic scenario model [26]; in the most optimistic scenario, we assumed that all HCC patients ”alive” at 17 months were ”cured”, and their death risk would be equal to their age-adjusted background mortality rate in the US (i.e., 3-year survival rate of 60.7%). We modeled the long-term outcome of sorafenib-treated patients based on the previously published literature, which resulted in less uncertainty when compared with that of atezo–bev [27]. (Details are shown in Supplementary Tables S1 and S2.)

### 2.2. Model Structure

This study was exempted from institutional review board approval. Our study follows the Consolidated Health Economic Evaluation Reporting Standards (CHEERS) reporting guideline for economic evaluations [28] (see Supplementary File).

We developed a Markov model to estimate the costs and the cost-effectiveness of first-line systemic treatments of HCC (Supplementary Materials: Supplementary Figure S1 and Technical Notes). Two first-line treatment options were evaluated: (A) atezo–bev vs. (B) sorafenib. We assumed that all treatments were continued until unacceptable toxicities or loss of clinical benefits. Following the failure of first-line treatment or unacceptable toxic effects, both groups would receive subsequent treatment until death. In the IMbrave 150 trial, 20.5% (69 of 336) and 44.2% (77 of 165) of patients in the atezo–bev and sorafenib arms, respectively, received subsequent systemic therapy [15].

Additionally, only direct medical costs were considered in our study. Each model cycle represented 3 weeks, and the time horizon was 5 years. Our model also applied a half-cycle correction. Furthermore, we adopted 3% annual discount rates for both the costs and health outcomes in our analysis. Primary outcomes of our study included total costs; life-years; quality-adjusted life-years (QALYs); and incremental cost-effectiveness ratios (ICERs), which was defined as the incremental costs divided by the incremental health outcomes. We considered two willingness-to-pay (WTP) thresholds: USD 100,000 per QALY and USD 150,000 per QALY. The model was implemented in TreeAge Pro 2020 (TreeAge Software, Williamstown, MA, USA), and statistical analyses were performed using R software version 3.5.1 (R core team) (Vienna, Austria).

### 2.3. Survival Estimates

The overall risk of death in any state was estimated as the probability of death from HCC as well as the background mortality rate from other causes in the US. For each treatment strategy (atezo–bev and sorafenib), the risk of death from HCC was estimated based on the OS curves of the published IMbrave 150 trial using the GetDataGraphDigitizer software package (version 2.25). Pseudo-individual patient data were generated with reference to Hoyle et al. [29,30]. We then fitted the reconstructed OS curves to Weibull distribution showing best fit when compared with exponential, log-logistic, and log-normal distributions, demonstrated by a lower value of Akaike Information Criterion (AIC) (Supplementary Figure S2).

We estimated the disease progression risk using the same approach by fitting the reconstructed PFS curves from the IMbrave 150 trial. For each group, the background mortality rate was based on the US life tables (Supplementary Table S1).

### 2.4. Utility Estimates

QALYs in our study were estimated by combining the survival time and a health utility (a health status value from 0 to 1 for death and perfect health, respectively), which was referred to as health-related quality of life (QOL). The European Organization for Research and Treatment of Cancer (EORTC) quality-of-life questionnaire for cancer (EORTC QLQ–C30) was used to access QOL in the IMbrave 150 trial [15]; however, only time to deterioration of global QOL, physical functioning, and role functioning domains were published; detailed patient-level data to approximate the European QOL 5-Dimension Questionnaire (EQ-5D) utilities were lacking. Therefore, we extrapolated QOL data from a recently published study evaluating atezo–bev in renal cell carcinoma and the previous health utility of sorafenib-treated HCC patients in first-line settings [21,31]. QOL utilities of 0.78 were assigned to both atezo–bev and sorafenib-treated patients [21,31]. Furthermore, we specified a utility weight of 0.68 for patients demonstrating disease progression [21]. We also considered utility decrements for each episode of developing grade 3 or 4 toxicities. Sensitivity analyses were conducted to address the uncertainties owing to assumptions.

### 2.5. Cost Estimates

Our primary analysis measured cost-effectiveness from a third-party payer perspective, although we assessed cost-effectiveness from a societal perspective in a scenario analysis [32]. Direct medical costs included the costs of drugs, administration, and management of adverse events. In addition to treatment-related mortality, we considered only grade 3–4 adverse effects that presented significantly different rates (≥3% difference) between treatment arms in the IMbrave 150 trial, which included hypertension, diarrhea, bilirubin increase, and hand–foot syndrome [15].

All patients who died in both arms were assumed to have received equivalent end-of-life (EOL) care [33]. The costs of post-progression therapy were calculated according to the number of patients receiving each post-progression drug and/or intervention listed in the IMbrave 150 trial [15]. To estimate the unit price of drugs, we used the 2020 average wholesale price and also incorporated the costs of drug administration from relevant sources [34,35,36,37]. The costs of local therapy were obtained from the 2020 Medicare Physician Fee Schedule [37]. Costs attributed to adverse effects were estimated based on the previous literature [38,39,40,41].

We adjusted all the above costs for inflation to reflect the 2020 USD value, using the US consumer price index. All information regarding the drug dose and the costs are listed in Table 1 and Supplementary Table S3.

### 2.6. Sensitivity Analyses

There are factors directly influencing the cost-effectiveness. To identify such factors, one-way deterministic sensitivity analyses were performed for each variable in our analysis. All variables varied over the plausible ranges, which were retrieved from their corresponding confidence intervals or by assuming a variance of 20% from base case values (Table 1). Furthermore, we conducted a probabilistic sensitivity analysis to assess the impact of uncertainty in all transition probabilities, costs, and health utilities using a Monte Carlo simulation with 100,000 iterations. We also considered the cost-effectiveness in all patient subgroups presented in the IMbrave 150 trial. Furthermore, we performed several scenario analyses to assess model sensitivity to long-term survival outcomes, duration of the immunotherapy combination, dosage of bevacizumab, and societal perspectives.

## 3. Results

### 3.1. Base Case Analysis

The model predicted that the life expectancy of patients receiving atezo–bev was 2.02 life-years, which was 0.51 life-years longer than that of patients receiving sorafenib (Table 2). After adjusting with QOL, patients receiving atezo–bev had 1.43 QALYs, which was 0.44 QALYs more than that of patients receiving sorafenib. Treatment with atezo–bev presented an additional cost of USD 79,074, resulting in an ICER of USD 155,047 per life-year, or USD 179,729 per QALY, when compared with that of patients receiving sorafenib.

### 3.2. Scenario and Sensitivity Analyses

The economic value of atezo–bev was strongly influenced by its long-term outcomes. Based on an optimistic survival assumption, atezo–bev resulted in an additional gain of 0.84 QALYs at USD 53,854 per QALY. At pessimistic survival assumption, atezo–bev resulted in an additional of 0.24 QALYs gain, with a cost of USD 385,857 per QALY. In the probabilistic sensitivity analyses, atezo–bev at optimistic assumption was cost-effective in 92.7% and 78.1% of simulations at WTP thresholds of USD 150,000 and 100,000, respectively; atezo–bev at base case was cost-effective in 45.6% and 24.5% of simulations at equivalent thresholds, whereas atezo–bev was cost-effective in only 9.7% and 4.3% of simulations, respectively, under the pessimistic assumption (Table 3, Supplementary Figure S3).

**Figure 1:**
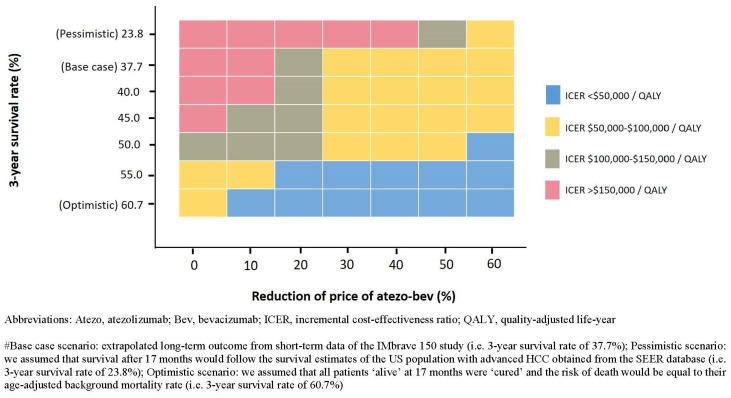
Results of two-way sensitivity analysis: price of the atezo–bev combination compared with the 3-year survival estimation.

Reducing the price of atezo–bev improved its cost-effectiveness, even if long-term outcomes were modest. At base case, a price reduction of 20% (atezo: USD 7535.33; bev: USD 94.08) and 29% (atezo: USD 6687.6; bev: USD 83.5) would satisfy the USD 150,000 and 100,000 per QALY WTP threshold, respectively. However, at pessimistic assumption, satisfying the USD 150,000 and 100,000 per QALY threshold would require price reductions of 47% (atezo: USD 4992.2; bev: USD 62.3) and 54% (atezo: USD 4332.8; bev: USD 54.1). At its current price, atezo–bev costs <USD 100,000 per QALY over a 3-year survival rate of approximately 55% and <USD 150,000 per QALY over a rate of approximately 50% (Figure 1).

**Figure 2:**
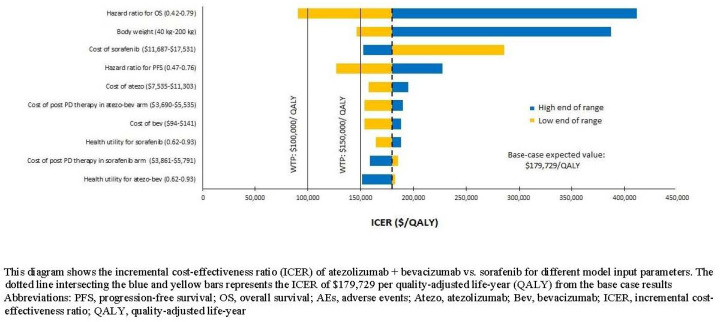
Results of the one-way sensitivity analysis (Tornado diagrams).

In one-way sensitivity analyses, our model was most sensitive to the HR for OS (0.58, range: 0.42–0.79) and patient body weight (70 kg, range: 40–200 kg). Several parameters presented moderate influences on the ICER: the price of atezo–bev, the price of sorafenib, HR of PFS, post-progression therapy costs, and health utilities (Figure 2).

In scenario analyses, when the duration of treatment was universally capped at ≤12 months or the dosage of bev reduced to ≤10 mg/kg, the atezo–bev combination would demonstrate cost-effectiveness at USD 150,000 per QALY threshold. The ICER of atezo–bev would modestly improve when the analyses considered each individual’s lifespan; however, considering societal perspectives would worsen its economic value (Table 4).

Following two-way sensitivity analyses, the results revealed that atezo–bev was cost-effective if the duration of treatment would be universally capped at ≤12 months or the dosage of bev was reduced to ≤10 mg/kg. However, under the long-term pessimistic survival estimation, atezo–bev was not cost-effective, regardless of the treatment duration or bev dosage. Furthermore, the ICERs were above USD 150,000 per QALY in nearly all utility and post-progression therapy cost combinations (Supplementary Figure S4).

Moreover, subgroup analyses reported that atezo–bev was most cost-effective in female patients, followed by patients with hepatitis C, Eastern cooperative group (ECOG) performance status 1, and Barcelona clinic liver cancer (BCLC) stage C (Supplementary Table S4).

## 4. Discussion

To the best of our knowledge, this study represents the most comprehensive cost-effectiveness analysis evaluating the combination of atezo–bev in HCC. Previous studies have suggested that atezo–bev is unlikely to be a cost-effective first line treatment of HCC [47,48]. In our model, atezo–bev provided survival gains in the advanced HCC population when compared with sorafenib; however, the economic value of this combination is highly dependent on its long-term clinical outcomes. At our most optimistic assumption that all patients alive at 17 months are “cured”, atezo–bev would result in remarkable survival gains (0.91 life-years and 0.84 QALYs) and good economic value (USD 53,584/QALY). However, the cost-effectiveness of atezo–bev progressively diminished as we modeled lower long-term survival rates. In the worst-case scenario modeling that patient survival beyond the study period followed the SEER data, atezo–bev would provide only modest survival benefits (0.21 life-years, 0.24 QALYs) and poor economic value (USD 385,857/QALY).

Atezo–bev represents a landmark breakthrough in HCC management as the first therapeutic strategy with demonstrated superiority over sorafenib. However, our results suggested that at the current price and treatment regime, its economic value remains uncertain. Moreover, our analyses showed that a price reduction might render payers more willing to bear the financial risk of uncertain long-term clinical outcomes. A moderate price reduction (20%) would allow the base case scenario to meet the WTP threshold of USD 150,000 per QALY. However, a substantial price reduction (47%) is needed for the modeled worst-case scenario. To justify the current price level, a 3-year survival rate of 50% is needed. When further expanded outcome data are reported, our model could provide a framework to adjust the price of atezo–bev in accordance with its long-term effectiveness.

Our model demonstrated sensitivity to treatment duration and bev dosage. Atezo–bev would consistently meet the WTP threshold of USD 150,000 per QALY when the atezo–bev use is universally capped at ≤12 months or/and the bev dosage is ≤10 mg/kg. In the IMbrave 150 trial, patients were treated until progression or unaccepted toxicities. The median duration of atezo and bev was 7.4 and 6.9 months, respectively; however, for responders who benefited from prolonged duration of treatment, the survival improvement and QALY gains from atezo–bev would lead to a parallel increase in treatment cost, which may offset its benefits from a value perspective. To date, the optimal duration of immune checkpoint inhibitors (ICI) or its combinations remain unknown; different durations have been used, ranging from until progression or toxicity, to a fixed duration of 2 years [38]. Despite the exploratory analysis of a 1-year fixed duration trial in non-small cell lung cancer unable to support the routine cap at 12 months [46], data from ICI-treated melanoma patients suggested that patients who achieved complete radiological response and received at least 6 months of therapy could benefit from durable remission [49].

Likewise, the optimal dosage of bev needs to be established. Studies comparing different doses are rare; the dosage of bev as approved by regulatory agents has been based on randomized studies that compared against placebos. Furthermore, the dose–effect relationship of bev has not been established in clinical practice; a low dose (7.5 mg/kg every 3 weeks) or high dose (10 mg/kg every 2 weeks or 15 mg/kg every 3 weeks) has been approved in the treatment of metastatic colorectal cancer and lung cancer. However, for other disease sites, including HCC, no comparative study has been undertaken to warrant dose reduction [50]. Our results demonstrated that optimization of the dosage scheme could permit the prescription of atezo–bev to a broader population, presenting a better cost-effectiveness ratio. To balance efficacy and financial toxicity, prospective studies and real-life data collection are warranted to delineate the optimal duration of therapy and bev dosage.

We did not compare the cost-effectiveness of lenvatinib, another approved first-line therapy of advanced HCC [3], against atezo–bev because of the lack of head-to-head trial data. However, previous studies have suggested that lenvatinib offered at least similar or even better efficacy and a more cost-effective therapy compared to sorafenib [51]. Both atezo–bev and lenvatinb have increased PFS compared to sorafenib, and the difference of their cost-effectiveness would be explainable by the cheaper price of lenvatinib. As a result, the cost associated with an increased duration of therapy would be minimized.

In recent years, several ICI-based treatments have been registered for treating advanced HCC based on early-phase data with limited follow-up. One strength of our study is that we addressed this uncertainty by modeling various scenarios by incorporating external information by assuming a “statistically cured” fraction, incorporating background mortality, and extrapolating registry data, to cover a broad range of plausible long-term outcomes for atezo–bev. Moreover, our results provide a framework for value evaluation when additional survival data are published. Of note, the updated overall survival data with median follow-up of 18 months were recently presented in an abstract form (median OS: 19.2 months vs. 13.4 months, HR 0.66) [52]. Based on our model, our conclusion would not alter with an ICER of USD 246,716 per QALY (95% CI USD 214,362 to USD 277,151); it was only cost effective in 28.0% and 14.6% of simulations at WTP thresholds of USD 150,000 and 100,000, respectively.

This study has several limitations. First, just as previous studies highlighted the differences of HCC patients enrolled into clinical trial vs. those in a real-world setting, for enrolment into the IMbrave 150 study, patients were required to present a well-compensated hepatic function and endoscopic evaluation within 6 months; those presenting high bleeding risks were excluded. As a result, a low rate of ≥ grade 3 bleeding was observed in the trial [15]. However, in real-life clinical settings, where timely endoscopic assessments may not always be feasible and with patients presenting broader liver functions are treated, the bleeding risks and the associated costs of AE management may increase, and the cost-effectiveness of atezo–bev would be further lowered.

Secondly, to date, no high quality long-term clinical outcome data for atezo–bev are available in HCC patients. We addressed this limitation by modeling long-term effectiveness scenarios, including the most optimistic assumption that all patients are “cured” if they survive beyond 17 months or the most pessimistic assumption that the survival rate was based on SEER data; although the two extreme scenarios are unlikely in real-life settings, the board range of survival rates covered by the two extremes was representative of plausible outcomes in patients clinically treated with atezo–bev. Additionally, we lacked long-term data on the sorafenib arm. However, sorafenib-treated patients are rarely cured; furthermore, we modeled the long-term survival rates on a recent meta-analysis [27], resulting in comparatively less uncertainty regarding the long-term outcomes of the sorafenib arm.

Thirdly, as with other modeling studies, our analysis was limited by data availability. We obtained cost estimates and health utility from other sources. We acknowledge that these estimations were not ideal; however, our model was insensitive to healthy utility value or costs other than those of upfront systemic treatments (Supplementary Figure S4). Several uncertain inputs may worsen the economic value of atezo–bev combinations, for example, the costs of post-progression therapy, as only half the patients in the atezo–bev arm experienced progression at the date of data cut-off. Lastly, we were unable to compare atezo–bev with another first-line therapy, lenvatinib, owing to a lack of robust head-to-head trial data.

## 5. Conclusions

The long-term effectiveness of atezo–bev is a critical but uncertain determinant of its cost-effectiveness. Price reduction would favorably influence its cost-effectiveness, even if the long-term clinical outcomes were modest. Further studies are warranted to optimize the duration and dosage of treatment.

## Figures and Tables

**Table 1:** Model parameters, baseline values, ranges, and distribution for sensitivity analyses.

Parameters	Base Case Value	Range		Reference	Distribution
		Minimum	Maximum		
Clinical effectiveness [15]					
HR for OS (atezo + bev vs.sorafenib)	0.58	0.42	0.79	[15]	Normal
HR for PFS (atezo + bev vs. sorafenib)	0.59	0.47	0.76	[15]	Normal
Weibull OS model with sorafenib	λ = 0.027, γ = 1.286	-	-	[15]	-
Weibull PFS model with sorafenib	λ = 0.093, γ = 1.312	-	-	[15]	-
Background mortality rate	Age-specific				
Rate of treatment discontinuation due to AEs [15]					
Atezo + Bev	0.07	0.056	0.084	[15]	Binomial
Sorafenib	0.10	0.08	0.12	[15]	Binomial
Proportion of patients with grade 3–4 AEs [15]					
Atezo + Bev					
Diarrhea	0.018	0.014	0.022	[15]	Binomial
Hand–foot syndrome	0.0	0.0	0.0	[15]	Binomial
Hypertension	0.152	0.122	0.182	[15]	Binomial
Increased bilirubin	0.024	0.019	0.029	[15]	Binomial
Sorafenib					
Diarrhea	0.051	0.041	0.061	[15]	Binomial
Hand–foot syndrome	0.083	0.066	0.1	[15]	Binomial
Hypertension	0.122	0.098	0.146	[15]	Binomial
Increased bilirubin	0.064	0.051	0.077	[15]	Binomial
Patient weight, kg	70	40	200	[42]	Triangular
Cost parameters, USD *					
Atezolizumab (1200 mg) (fixed) (every 3 weeks)	9419.16	7535.33	11,302.99	[4,5]	Triangular
Bevacizumab (15 mg/kg) (every 3 weeks)	117.60	94.08	141.12	[34,35,36]	Triangular
Sorafenib (every 3 weeks)	14,609.28	11,687.42	17,531.14	[34,35,36]	Triangular
Drug administration (every 3 weeks)	435.04	348.03	522.05	[34,35,36]	Triangular
CT imaging (every 6 weeks)	1543	1235	1852	[35]	Triangular
Other care (every week)	174.5	139.6	209.4	[20]	Triangular
In-patient EOL care	7360.16	6208.85	9313.77	[31]	Triangular
Post-progression therapy cost per cycle in atezo + bev	4612.66	3690.13	5535.20	[15]	Triangular
Post-progression therapy cost per cycle in sorafenib	4825.93	3860.74	5791.11	[15]	Triangular
Societal costs, USD *					
Caregiver (every 3 weeks)	382.21	305.77	458.65	[43]	Triangular
Patient time	896.2	716.96	1075.44	[43,44]	Triangular
Parking/meals/travel	329.5	263.6	395.4	[43,45]	Triangular
Management of Grade 3–4 AEs, USD *					
Diarrhea	88.38	70.70	106.05	[38,39,40,41]	Triangular
Hand–foot syndrome	145.65	116.51	174.78	[38,39,40,41]	Triangular
Hypertension	64.01	51.21	76.81	[38,39,40,41]	Triangular
Increased bilirubin	0	0	0	Estimates	Triangular
Utilities and dis-utilities					
In atezo–bev first-line therapy	0.78	0.624	0.936	[31]	Triangular
In sorafenib first-line therapy	0.78	0.624	0.936	[21]	Triangular
In second-line therapy	0.68	0.54	0.82	[21]	Triangular
Dis-utilities					
Diarrhea	−0.103	−0.082	−0.123	[46]	Triangular
Hand–foot syndrome	−0.116	−0.093	−0.139	[46]	Triangular
Hypertension	−0	−0	−0	[46]	Triangular
Increased bilirubin	−0	−0	−0	[46]	Triangular

Abbreviations: atezo, atezolizumab; bev, bevacizumab; OS, overall survival; PFS, progression-free survival; AE, adverse event; CT, computed tomography; DM, diabetics mellitus; EOL, end-of-life; HR, hazard ratio. * Adjustment of the costs for inflation to reflect the 2020 USD value, using the US consumer price index.

**Table 2:** Summary of base case results.

Parameters	Atevo + Bev	Sorafenib
Treatment duration, months	Atezo: 7.4; Bev: 6.9	2.8
Cost, USD		
Total cost	713,742	634,668
Clinical effectiveness, months		
Median PFS	6.8	4.3
Median OS	NE	13.2
Median post-progression survival	NE	8.9
Utilities		
Utility while on first-line treatment	0.78	0.78
AEs	−0.013	−0.013
Utility at progression	0.68	0.68
QALYs	1.426	0.987
QALY gain	0.440	
Life-years	2.02	1.51
Incremental life-year	0.51	
ICER, USD		
Per life-year	155,047 (142,953–174,815) *	
Per QALY	179,729 (163,932–191,054) *	

Abbreviations: atezo + bev: atezolizumab + bevacizumab; AEs, adverse events; ICER, incremental cost-effectiveness ratio; OS, overall survival; PFS, progression-free survival; QALY, quality-adjusted life-year. * Values in parentheses represent 95% credible intervals derived from the results of probabilistic sensitivity analyses.

**Table 3:** Clinical and economic outcomes of three effectiveness scenarios are shown: (a) base case; (b) pessimistic survival estimation; (c) optimistic survival estimation.

					Cost-Effectiveness Acceptability at WTP, %	
Scenario	Life-Years	QALYs	Cost, 2020 US USD	ICER, USD/QALY	USD 100,000/ QALY	USD 150,000/ QALY
Atezo–bev						
Base case *	2.02	1.426	713,742	179,729	24.5	45.6
Pessimistic survival *	1.78	1.227	727,557	385,857	4.3	9.7
Optimistic survival *	2.48	1.825	679,811	53,854	78.1	92.7
Sorafenib	1.51	0.987	634,668	NA	NA	NA

Results of the probabilistic sensitivity analyses based on 100,000 Monte Carlo simulations, which involves sampling model variable values from distribution imposed on variables, to indicate uncertainty whether the atezolizumab + bevacizumab combination is cost-effective at different willingness-to-pay thresholds. * Base case scenario: extrapolated long-term outcome from short-term data of IMbrave 150 study (i.e., 3-year survival rate of 37.7%); pessimistic scenario: we assumed that survival after 17 months would follow the survival estimates of the US population with advanced HCC obtained from the SEER database (i.e., 3-year survival rate of 23.8%); optimistic scenario: we assumed that all patients ”alive” at 17 months were ”cured” and the risk of death would be equal to their age-adjusted background mortality rate (i.e., 3-year survival rate of 60.7%). Abbreviations: atezo, atezolizumab; bev, bevacizumab; EOL, end-of-life; ICER, incremental cost-effectiveness ratio; QALY, quality-adjusted life-yea; WTP, willingness-to-pay.

**Table 4:** Summary of selected scenario analyses.

Parameters	ICER (USD /QALY)
Base case analysis	179,729
Model Perspective	
Third-party perspective (base case)	179,729
Societal perspective	219,058
Scenario analysis	
Duration of atezo–bev	
Until disease progression (base case)	179,729
Max 24 months	176,626
Max 18 months	167,058
Max 12 months	136,205
Max 6 months	18,598
Dosage of bev (assuming a body weight of 70 kg)	
15 mg/kg (base case)	179,729
12.5 mg/kg	167,405
10 mg/kg	122,286
7.5 mg/kg	107,969
5 mg/kg	81,952
Time horizon	
5 years (base case)	179,729
10 years	156,988
Lifetime	156,710

Abbreviations: ICER, incremental cost-effectiveness ratio; atezo, atezolizumab; bev, bevacizumab; QALY, quality-adjusted life-year; OS, overall survival; HCC, hepatocellular carcinoma.

## Data Availability

The data that support the findings of this study are available from the corresponding author, upon reasonable request.

## Supplementary Materials

The following are available online at https://www.mdpi.com/2072-6694/13/5/931/s1, CHEERS Checklist. Technical notes 1: Justify the use of Markov survival model. Table S1: Background mortality rate. Table S2: Survival estimates for atezolizumab–bevacizumab and sorafenib. Table S3: Drug dose and costs. Table S4: Results of subgroup analyses. Figure S1: Simplified Markov model. Figure S2: Quantitative measures of goodness of fit using AIC: Comparison of reconstructed survival modeling. Figure S3: Cost-effectiveness acceptability curves. Figure S4: Results of two-way sensitivity analyses.

## References

[1] Bray F., Ferlay J., Soerjomataram I., Siegel R.L., Torre L.A., Jemal A. (2018). Global cancer statistics 2018: GLOBOCAN estimates of incidence and mortality worldwide for 36 cancers in 185 countries. CA Cancer J. Clin..

[2] Llovet J.M., Ricci S., Mazzaferro V., Hilgard P., Gane E., Blanc J.F., de Oliveira A.C., Santoro A., Raoul J.L., Forner A. (2008). Sorafenib in advanced hepatocellular carcinoma. N. Engl. J. Med..

[3] Kudo M., Finn R.S., Qin S., Han K.H., Ikeda K., Piscaglia F., Baron A., Park J.W., Han G., Jassem J. (2018). Lenvatinib versus sorafenib in first-line treatment of patients with unresectable hepatocellu-lar carcinoma: A randomised phase 3 non-inferiority trial. Lancet.

[4] Zhu A.X., Finn R.S., Edeline J., Cattan S., Ogasawara S., Palmer D., Verslype C., Zagonel V., Fartoux L., Vogel A. (2018). Pembrolizumab in patients with advanced hepatocellular carcinoma previously treated with sorafenib (KEYNOTE-224): A non-randomised, open-label phase 2 trial. Lancet Oncol..

[5] El-Khoueiry A.B., Sangro B., Yau T., Crocenzi T.S., Kudo M., Hsu C., Kim T.Y., Choo S.P., Trojan J., Welling T.H. (2017). Nivolumab in patients with advanced hepatocellular carcinoma (CheckMate 040): An open-label, non-comparative, phase 1/2 dose escalation and expansion trial. Lancet.

[6] Finn R.S., Ryoo B.Y., Merle P., Kudo M., Bouattour M., Lim H.Y., Breder V., Edeline J., Chao Y., Ogasawara S. (2020). Pembrolizumab As Second-Line Therapy in Patients with Advanced Hepatocellular Carci-noma in KEYNOTE-240: A Randomized, Double-Blind, Phase III Trial. J. Clin. Oncol..

[7] Yau T., Park J.W., Finn R.S., Cheng A.L., Mathurin P., Edeline J., Kudo M., Han K.H., Hardling J.J., Merle P. (2019). CheckMate 459: A Randomized, Multi-Center Phase 3 Study of Nivolumab (NIVO) vs Soraf-enib (SOR) as First-Line (1L) Treatment in Patients (pts) With Advanced Hepatocellular Carcinoma (aHCC). Ann. Oncol..

[8] Chen D.S., Hurwitz H. (2018). Combinations of Bevacizumab with Cancer Immunotherapy. Cancer J..

[9] Herbst R.S., Soria J.C., Kowanetz M., Fine G.D., Hamid O., Gordon M.S., Sosman J.A., McDermott D.F., Powderly J.D., Gettinger S.N. (2014). Predictive correlates of response to the anti-PD-L1 antibody MPDL3280A in cancer patients. Nature.

[10] Morse M.A., Sun W., Kim R., He A.R., Abada P.B., Mynderse M., Finn R.S. (2019). The Role of Angiogenesis in Hepatocellular Carcinoma. Clin. Cancer Res..

[11] Zhu A.X., Duda D.G., Sahani D.V., Jain R.K. (2011). HCC and angiogenesis: Possible targets and future directions. Nat. Rev. Clin. Oncol..

[12] Wallin J.J., Bendell J.C., Funke R., Sznol M., Korski K., Jones S., Hernandez G., Mier J., He X., Hodi F.S. (2016). Atezolizumab in combination with bevacizumab enhances antigen-specific T-cell mi-gration in metastatic renal cell carcinoma. Nat. Commun..

[13] Hegde P.S., Wallin J.J., Mancao C. (2018). Predictive markers of anti-VEGF and emerging role of angiogenesis inhibitors as immuno-therapeutics. Semin. Cancer Biol..

[14] Lee M., Ryoo B., Hsu C., Numata K., Stein S., Verret W., Hack S., Spahn J., Liu B., Abdullah H. (2019). Randomised Efficacy and Safety Results for Atezolizumab (Atezo) + Bevacizumab (Bev) in Pa-tients (pts) With Previously Untreated, Unresectable Hepatocellular Carcinoma (HCC). Ann. Oncol..

[15] Finn R.S., Qin S., Ikeda M., Galle P.R., Ducreux M., Kim T.Y., Kudo M., Breder V., Merle P., Kaseb A.O. (2020). Atezolizumab plus Bevacizumab in Unresectable Hepatocellular Carcinoma. N. Engl. J. Med..

[16] Casak S.J., Donoghue M., Fashoyin-Aje L., Jiang X., Rodriguez L., Shen Y.L., Xu Y., Jiang X., Liu J., Zhao H. (2020). FDA Approval Summary: Atezolizumab Plus Bevacizumab for the Treatment of Patients with Advanced Unresectable or Metastatic Hepatocellular Carcinoma. Clin. Cancer Res..

[17] Yang J.D., Hainaut P., Gores G.J., Amadou A., Plymoth A., Roberts L.R. (2019). A global view of hepatocellular carcinoma: Trends, risk, prevention and management. Nat. Rev. Gastroenterol. Hepatol..

[18] Yang X., Wang D., Lin J., Yang X., Zhao H. (2020). Atezolizumab plus bevacizumab for unresectable hepatocellular carcinoma. Lancet Oncol..

[19] Parikh N.D., Singal A.G., Hutton D.W. (2017). Cost-effectiveness of regorafenib as second-line therapy for patients with advanced hepatocellular carcinoma. Cancer.

[20] Soto-Perez-de-Celis E., Aguiar P.N., Cordon M.L., Chavarri-Guerra Y., Lopes G.L. (2019). Cost-effectiveness of cabozantinib in the second-line treatment of advanced hepatocellular carcinoma. J. Natl. Compr. Cancer Netw..

[21] Cammà C., Cabibbo G., Petta S., Enea M., Iavarone M., Grieco A., Gasbarrini A., Villa E., Zavaglia C., Bruno R. (2013). Cost-effectiveness of sorafenib treatment in field practice for patients with hepatocellular carcinoma. Hepatology.

[22] Chiang C.L., Chan S.K., Lee S.F., Wong I.O., Choi H.C. (2021). Cost-effectiveness of Pembrolizumab as a Second-Line Therapy for Hepatocellular Carcinoma. JAMA Netw. Open.

[23] Othus M., Bansal A., Koepl L., Wagner S., Ramsey S. (2017). Accounting for Cured Patients in Cost-Effectiveness Analysis. Value Health.

[24] Bullement A., Latimer N.R., Bell Gorrod H. (2019). Survival Extrapolation in Cancer Immunotherapy: A Validation-Based Case Study. Value Health.

[25] Prasad V., Mailankody S. (2015). How should we assess the value of innovative drugs in oncology? Lessons from cost-effectiveness analyses. Blood.

[26] Surveillance, Epidemiology, and End Results (SEER) Program (2020). SEER*Stat Database: Incidence—SEER 9 Regs Research Data. Linked to County Attributes—Total U.S., 1975–2017 Counties, National Cancer Institute, DCCPS, Surveillance Research Program. https://seer.cancer.gov.

[27] Cabibbo G., Cucchetti A., Cammà C., Casadei-Gardini A., Celsa C., Emanuele Maria Rizzo G., Johnson P., Ercolani G. (2019). Outcomes of hepatocellular carcinoma patients treated with sorafenib: A meta-analysis of Phase III trials. Future Oncol..

[28] Husereau D., Drummond M., Petrou S., Carswell C., Moher D., Greenberg D., Augustovski F., Briggs A.H., Mauskopf J., Loder E. (2013). Consolidated Health Economic Evaluation Reporting Standards (CHEERS)—Explanation and elaboration: A report of the ISPOR Health Economic Evaluation Publication Guidelines Good Reporting Practices Task Force. Value Health.

[29] Hoyle M.W., Henley W. (2011). Improved curve fits to summary survival data: Application to economic evaluation of health tech-nologies. BMC Med. Res. Methodol..

[30] Wan X., Peng L., Li Y. (2015). A Review and Comparison of Methods for Recreating Individual Patient Data from Published Kaplan-Meier Survival Curves for Economic Evaluations: A Simulation Study. PLoS ONE.

[31] Atkins M.B., Rini B.I., Motzer R.J., Powles T., McDermott D.F., Suarez C., Bracarda S., Stadler W.M., Donskov F., Gurney H. (2020). Patient-Reported Outcomes from the Phase III Randomized IMmotion151 Trial: Ate-zolizumab + Bevacizumab versus Sunitinib in Treatment-Naïve Metastatic Renal Cell Carcinoma. Clin. Cancer Res..

[32] Sanders G.D., Neumann P.J., Basu A., Brock D.W., Feeny D., Krahn M., Kuntz K.M., Meltzer D.O., Owens D.K., Prosser L.A. (2016). Recommendations for Conduct, Methodological Practices, and Reporting of Cost-effectiveness Analyses: Second Panel on Cost-Effectiveness in Health and Medicine. JAMA.

[33] May P., Normand C., Cassel J.B., Del Fabbro E., Fine R.L., Menz R., Morrison C.A., Penrod J.D., Robinson C., Morrison R.S. (2018). Economics of palliative care for hospitalized adults with serious illness: A meta-analysis. JAMA Intern. Med..

[34] Academy of Managed Care Pharmacy (2019). Guide to Pharmaceutical Payment Methods.

[35] Centers for Medicare & Medicaid Services: 2020 ASP Drug Pricing Files. https://www.cms.gov/medicare/medicare-part-b-drug-average-sales-price/2020-asp-drug-pricing-files.

[36] U.S. Department of Veterans Affairs (2020). Office of Procurement, Acquisition and Logistics (OPAL) Price Data.

[37] U.S. Centers for Medicare & Medicaid Services Physician Fee Schedule: CY 2020 Physician Fee Schedule Final Rule. https://www.cms.gov/Medicare/Medicare-Fee-for-Service-Payment/PhysicianFeeSched/.

[38] Wong W., Yim Y.M., Kim A., Cloutier M., Gauthier-Loiselle M., Gagnon-Sanschagrin P., Guerin A. (2018). Assessment of costs associated with adverse events in patients with cancer. PLoS ONE.

[39] Goldstein D.A., Ahmad B.B., Chen Q., Ayer T., Howard D.H., Lipscomb J., El-Rayes B.F., Flowers C.R. (2015). Cost-Effectiveness Analysis of Regorafenib for Metastatic Colorectal Cancer. J. Clin. Oncol..

[40] Criss S.D., Mooradian M.J., Watson T.R., Gainor J.F., Reynolds K.L., Kong C.Y. (2019). Cost-effectiveness of Atezolizumab Combination Therapy for First-Line Treat-ment of Metastatic Nonsquamous Non–Small Cell Lung Cancer in the United States. JAMA Netw. Open.

[41] Wan X., Luo X., Tan C., Zeng X., Zhang Y., Peng L. (2019). First-line atezolizumab in addition to bevacizumab plus chemotherapy for metastatic, nonsquamous non-small cell lung cancer: A United States-based cost-effectiveness analysis. Cancer.

[42] Wan X., Zhang Y., Tan C., Zeng X., Peng L. (2019). First-line Nivolumab Plus Ipilimumab vs Sunitinib for Metastatic Renal Cell Carcinoma: A Cost-effectiveness Analysis. JAMA Oncol..

[43] Li C., Zeliadt S.B., Hall I.J., Smith J.L., Ekwueme D.U., Moinpour C.M., Penson D.F., Thompson I.M., Keane T.E., Ramsey S.D. (2013). Burden among partner caregivers of patients diagnosed with localized prostate cancer within 1 year after diagnosis: An economic perspective. Support Care Cancer.

[44] Hopkins R.B., Goeree R., Longo C.J. (2010). Estimating the national wage loss from cancer in Canada. Curr. Oncol..

[45] De Almeida J.R., Moskowitz A.J., Miles B.A., Goldstein D.P., Teng M.S., Sikora A.G., Gupta V., Posner M., Genden E.M. (2016). Cost-effectiveness of transoral robotic surgery versus (chemo)radiotherapy for early T classification oropharyngeal carcinoma: A cost-utility analysis. Head Neck.

[46] Spigel D.R., McLeod M., Hussein M.A., Waterhouse D.M., Einhorn L., Horn L., Creelan B., Babu S., Leighl N.B., Couture F. (2017). Randomized results of fixed-duration (1-yr) vs continuous nivolumab in patients (pts) with advanced non-small cell lung cancer (NSCLC). Ann. Oncol..

[47] Hou Y., Wu B. (2020). Atezolizumab plus bevacizumab versus sorafenib as first-line treatment for unresectable hepatocellular carcinoma: A cost-effectiveness analysis. Cancer Commun..

[48] Dubois de Gennes C., Mazaleyrat B., Sanchez Alvarez J., Cawston H. (2020). Preliminary Results of a Cost Effectiveness MODEL of Atezolizumab PLUS Bevacizumab in Unresectable Hepatocellular Carcinoma (HCC) in France. Value Health.

[49] Jansen Y.J.L., Rozeman E.A., Mason R., Goldinger S.M., Foppen M.G., Hoejberg L., Schmidt H., Van Thienen J.V., Haanen J.B.A.G., Tiainen L. (2019). Discontinuation of anti-PD-1 antibody therapy in the absence of disease progres-sion or treatment limiting toxicity: Clinical outcomes in advanced melanoma. Ann. Oncol..

[50] Falk A.T., Barrière J., François E., Follana P. (2015). Bevacizumab: A dose review. Crit. Rev. Oncol. Hematol..

[51] Kim J.J., McFarlane T., Tully S., Wong W.W.L. (2020). Lenvatinib versus Sorafenib as First-Line Treatment of Unresectable Hepatocellular Carcinoma: A Cost-Utility Analysis. Oncologist.

[52] Finn R.S., Qin S., Ikeda M., Galle P.R., Ducreux M., Kim T.Y., Lim H.Y., Kudo M., Breder V.V., Merle P. (2021). IMbrave150: Updated overall survival (OS) data from a global, randomized, open-label phase III study of atezolizumab (atezo) + bevacizumab (bev) vs sorafenib (sor) in patients (pts) with unresectable hepatocellular carcinoma (HCC). J. Clin. Oncol..

