# Supplementary material for: First-Line Atezolizumab Plus Bevacizumab versus Sorafenib in Hepatocellular Carcinoma: A Cost-Effectiveness Analysis
Source: Cancers (Basel). 2021 Feb 24;13(5):931. doi: 10.3390/cancers13050931 (PMC7956424; doi:10.3390/cancers13050931)
Supplement: Supplementary file 1 [file cancers-13-00931-s001.pdf]

First-Line Atezolizumab Plus Bevacizumab versus Sorafenib in Hepatocellular Carcinoma: A Cost-Effectiveness Analysis

Chi-Leung Chiang, Sik-Kwan Chan, Shing-Fung Lee and Horace Cheuk-Wai Choi

CHEERS Checklist

Technical notes 1: Justify the use of Markov survival model

CHEERS checklist—Items to include when reporting economic evaluations of health interventions

Section/Item	Item No	Recommendation	Reported on Page No/ line No
		**Title and abstract**	
Title	1	Identify the study as an economic evaluation or use more specific terms such as "cost-effectiveness analysis", and describe the interventions compared.	1
Abstract	2	Provide a structured summary of objectives, perspective, setting, methods (including study design and inputs), results (including base case and uncertainty analyses), and conclusions.	3-4
		**Introduction**	
Background and objectives	3	Provide an explicit statement of the broader context for the study.	5-6
		Present the study question and its relevance for health policy or practice decisions.	5-6
		**Methods**	
Target population and subgroups	4	Describe characteristics of the base case population and subgroups analysed, including why they were chosen.	6
Setting and location	5	State relevant aspects of the system(s) in which the decision(s) need(s) to be made.	6
Study perspective	6	Describe the perspective of the study and relate this to the costs being evaluated.	7
Comparators	7	Describe the interventions or strategies being compared and state why they were chosen.	6
Time horizon	8	State the time horizon(s) over which costs and consequences are being evaluated and say why appropriate.	7
Discount rate	9	Report the choice of discount rate(s) used for costs and outcomes and say why appropriate.	7
Choice of health outcomes	10	Describe what outcomes were used as the measure(s) of benefit in the evaluation and their relevance for the type of analysis performed.	7
Measurement of effectiveness	11a	*Single study-based estimates:* Describe fully the design features of the single effectiveness study and why the single study was a sufficient source of clinical effectiveness data.	7-8
	11b	*Synthesis-based estimates*: Describe fully the methods used for identification of included studies and synthesis of clinical effectiveness data.	
Measurement and valuation of preference based outcomes	12	If applicable, describe the population and methods used to elicit preferences for outcomes.	not applicable
Estimating resources and costs	13a	*Single study-based economic evaluation:*Describe approaches used to estimate resource use associated with the alternative interventions. Describe primary or secondary research methods for valuing each resource item in terms of its unit cost. Describe any adjustments made to approximate to opportunity costs.	

Section/Item	Item No	Recommendation	Reported on Page No/ line No
	13b	*Model-based economic evaluation:* Describe approaches and data sources used to estimate resource use associated with model health states. Describe primary or secondary research methods for valuing each resource item in terms of its unit cost. Describe any adjustments made to approximate to opportunity costs.	8-9
Currency, price date, and conversion	14	Report the dates of the estimated resource quantities and unit costs. Describe methods for adjusting estimated unit costs to the year of reported costs if necessary. Describe methods for converting costs into a common currency base and the exchange rate.	9-10
-10Choice of model	15	Describe and give reasons for the specific type of decision-analytical model used. Providing a figure to show model structure is strongly recommended.	7
Assumptions	16	Describe all structural or other assumptions underpinning the decision-analytical model.	8
Analytical methods	17	Describe all analytical methods supporting the evaluation. This could include methods for dealing with skewed, missing, or censored data; extrapolation methods; methods for pooling data; approaches to validate or make adjustments (such as half cycle corrections) to a model; and methods for handling population heterogeneity and uncertainty.	7-9
**Results**			
Study parameters	18	Report the values, ranges, references, and, if used, probability distributions for all parameters. Report reasons or sources for distributions used to represent uncertainty where appropriate. Providing a table to show the input values is strongly recommended.	Table 1
33Incremental costs and outcomes	19	For each intervention, report mean values for the main categories of estimated costs and outcomes of interest, as well as mean differences between the comparator groups. If applicable, report incremental cost-effectiveness ratios.	10, Table 2
Characterising uncertainty	20a	*Single study-based economic evaluation:*Describe the effects of sampling uncertainty for the estimated incremental cost and incremental effectiveness parameters, together with the impact of methodological assumptions (such as discount rate, study perspective).	
	20b	*Model-based economic evaluation:* Describe the effects on the results of uncertainty for all input parameters, and uncertainty related to the structure of the model and assumptions.	10-12
Characterising heterogeneity	21	If applicable, report differences in costs, outcomes, or cost-effectiveness that can be explained by variations between subgroups of patients with different baseline characteristics or other observed variability in effects that are not reducible by more information.	11, supplementary Table 4
**Discussion**			
Study findings, limitations, generalisability, and current knowledge	22	Summarise key study findings and describe how they support the conclusions reached. Discuss limitations and the generalisability of the findings and how the findings fit with current knowledge.	13-17
**Other**			
Source of funding	23	Describe how the study was funded and the role of the funder in the identification, design, conduct, and reporting of the analysis. Describe other non-monetary sources of support.	2
Conflicts of interest	24	Describe any potential for conflict of interest of study contributors in accordance with journal policy. In the absence of a journal policy, we recommend authors comply with International Committee of Medical Journal Editors recommendations.	2

For consistency, the CHEERS statement checklist format is based on the format of the CONSORT statement checklist.

Technical notes 1. Justifying the choice of Markov Modeling Over Partitioned Survival Modeling

The IMbrave 150 trial has reported outcomes as co-primary endpoints of overall survival (OS) and progression-free survival (PFS) curves. These two curves contain combined information on the rates of three clinical outcomes: disease progression, pre-progression mortality, and post-progression mortality. Notably, accurate oncological modeling depends on accurate identification of time-dependent rates of these three distinct rates from the published survival curves.

The Markov survival model (MSM) and partitioned survival model (PSM) are two commonly used models in oncological modeling. We select the MSM model in this analysis owing to the following reasons. First, PSM assumes constant costs and utilities over time, which is inappropriate for modeling advanced HCC patients. Second, PSM does not accurately model multiple lines of therapy. However, in the IMbrave 150 study, a substantial number of patients received two or more lines of systemic therapies or additional local therapy.

Table S1. Background mortality rate. Estimates of background mortality rate for each age are provided in the US life Supplementary Table; Arias E, Heron M, Xu J. United States Life Supplementary Tables, 2019. Natl Vital Stat Rep. 2019; 68:1-65.

Age (years)	Background Mortality rate	Age (Years)	Background Mortality Rate	Age (Years)	Background Mortality Rate
18	0.000603	57	0.008857	96	0.291442
19	0.000698	58	0.009542	97	0.314700
20	0.000795	59	0.010285	98	0.338142
21	0.000889	60	0.011098	99	0.361537
22	0.000970	61	0.011952	100	1
23	0.001424	62	0.012814		
24	0.001497	63	0.013657		
25	0.001561	64	0.014502		
26	0.001624	65	0.015384		
27	0.001682	66	0.016444		
28	0.001737	67	0.017624		
29	0.001792	68	0.018968		
30	0.001847	69	0.019586		
31	0.001900	70	0.022109		
32	0.001952	71	0.024359		
33	0.002003	72	0.026347		
34	0.002053	73	0.028810		
35	0.002111	74	0.031309		
36	0.002174	75	0.034486		
37	0.002233	76	0.038026		
38	0.002285	77	0.042286		
39	0.002340	78	0.046547		
40	0.002413	79	0.051534		
41	0.002516	80	0.057008		
42	0.002649	81	0.062923		
43	0.002811	82	0.069911		
44	0.002999	83	0.078099		
45	0.003203	84	0.086754		
46	0.003433	85	0.096549		
47	0.003709	86	0.106472		
48	0.004047	87	0.119677		
49	0.004445	88	0.134128		
50	0.004874	89	0.149846		
51	0.005331	90	0.166829		

52	0.005844	91	0.185047	
53	0.006408	92	0.204441	
54	0.007003	93	0.224919	
55	0.007607	94	0.246354	
56	0.008219	95	0.26890	

Table S2. Survival Estimates for atezolizumab + bevacizumab and sorafenib.

	Survival (%)					
**Atezo-Bev**	**6 months #**	**12 months #**	**2 years +**	**3 years +**	**4 years +**	**5 years**
Base case *	84.8 (80.9-88.7)	67.2 (61.3-73.1)	**51.8% (46.5%-56.7%)**	**37.7% (32.4%-41.2%)**	25.9% (22.6%-30.0%)	19.8% (17.2%-21.8%)
Pessimistic survival *			**33.1% (28.2%-38.4%)**	**23.8% (19.2%-28.6%)**	17.5% (12.1%-21.0%)	13.6% (6.8%-16.6%)
Optimistic survival *			**61.8% (56.2%-67.7%)**	**60.7% (55.1%-66.5%)**	59.6% (54.1%-65.2%)	58.6% (53.2%-64.0%)
Sorafenib	72.2 (65.1-79.4)	54.6 (45.2-64.0)	**24.0% (22.5%-25.8%)**	**17.3% (15.5%-19.5%)**	15.1% (11.8%-19.4%)	NA

* Base case scenario: extrapolated long-term outcome from short-term data of the IMbrave 150 study (i.e. 3-year survival rate of 37.7%); Pessimistic scenario: we assumed the survival after 17 months would follow the survival estimates of the US population with advanced HCC obtained from the SEER database (i.e. 3-year survival rate of 23.8%); Optimistic scenario: we assumed that all patients 'alive' at 17 months were 'cured' and the risk of death would be equal to their age-adjusted background mortality rate (i.e. 3-year survival rate of 60.7%). # The 6-month and 12-month survival rates were based on IMbrave 150 data. + The 2-year and 3-year survival estimates of atezo-bev were estimated based on assumptions of base case, optimistic, and pessimistic scenarios; survival estimate for sorafenib was modeled based on previous literature [20] Abbreviations: Atezo, atezolizumab; Bev, bevacizumab; EOL, end-of-life; ICER, incremental cost-effectiveness ratio; QALY, quality-adjusted life-year; WTP, willingness-to-pay; NA, not available.

Table S3. Drug dose and costs. Base case: 70 kg, body surface area 1.86 m^2.

Drug	Dose	Unit price ($)	Cost for 1 model cycle ($, 3 weeks)
**Systemic therapy**			
Atezolizumab + Bevacizumab	Atezo 1200 mg on day 1<br>Bev 15 mg per kg on day 1 every 3 weeks	7.85/mg (Atezo)<br>7.84/mg (Bev)	9419.2 + 8,232 = 17,651.2
Sorafenib	400 mg twice daily	0.87/mg	14,609.3
Lenvatinib	12 mg daily	55.19/mg	13,907.2
Regorafenib	160 mg daily for 21 days every 4 weeks	3.99/mg	10,049.1
Cabozatinib	60 mg daily	9.64/mg	12,150.0
Ramucirumab	8 mg per Kg every 2 weeks	12.213/mg	10,258.9
Nivolumab	3 mg per Kg every 2 weeks or<br>240 mg every 2 weeks	28.534/mg	10272.2
Pembrolizumab	200 mg every 3 weeks	50.927/mg	10,185.4
FOLFOX	Oxa 85 mg/m^2 intravenously [IV] on day 1; LV 200 mg/m^2 IV from hour 0 to 2 on days 1 and 2; and FU 400 mg/m^2 IV bolus at hour 2, then 600 mg/m^2 over 22 h on days 1 and 2, once every 2 weeks	107.548/ two weekly cycle	161.3
**Intervention**			
Radiofrequency ablation	NA	4,833	NA

Trans-arterial embolization	NA	9,908	NA
Trans-arterial chemoembolization	NA	9,908	NA
Trans-catheter arterial infusion	NA	2,771	NA
Trans-arterial radio-embolization	NA	10,145	NA

Abbreviation: Atezo: atezolizumab; Bev: bevacizumab; Oxa: oxaliplatin; LV: leucovorin; NA: not available.

Table S4. Results of the subgroup analysis.

Subgroup	Sample size		HR for PFS (95% Cl)	HR for OS (95% Cl)	ICER ($/QALY) (95% Cl)	Cost-effectiveness acceptability at WTP	
	Atezo + Avastin N = 336	Sorafenib N = 165				$100,000/ QALY	$150,000 /QALY
**Sex**							
Male	277	137	0.59 (0.45-0.77)	0.66 (0.47-0.92)	246705 (131833-284463)	10.3%	24.1%
Female	59	28	0.60 (0.34-1.06)	0.35 (0.15-0.81)	59852 (46890-79101)	76.2%	92.9%
**Geographic location**							
Asia without Japan	133	68	0.46 (0.31-0.67)	0.53 (0.32-0.87)	196386 (178712-228593)	20.5%	37.3%
Non-Asia with Japan	203	97	0.70 (0.52-0.96)	0.65 (0.44-0.98)	194260 (193558-200662)	23.8%	39.4%
**ECOG**							
0	209	103	0.57 (0.42-0.78)	0.67 (0.43-1.06)	249985 (155262-264125)	11.7%	23.5%
1	127	62	0.63 (0.44-0.91)	0.51 (0.33-0.80)	121189 (114416-122858)	43.6%	62.6%
**AFP (ng/ml)**							
<400	210	104	0.49 (0.36-0.66)	0.52 (0.34-0.81)	177493 (161317-183713)	24.4%	43.4%
≥400	126	61	0.79 (0.54-1.16)	0.68 (0.43-1.08)	197132 (164766-350982)	23.4%	39.3%
**EHS and/or MVI**							
Yes	258	120	0.53 (0.41-0.70)	0.55 (0.39-0.77)	181514 (133262-217135)	23.1%	45.2%
No	78	45	0.72 (0.42-1.24)	0.69 (0.29-1.65)	229874 (217476-330464)	19.5%	32.2%
**Viral status**							
HBV	164	76	0.47 (0.33-0.67)	0.51 (0.32-0.81)	179330 (162663-187810)	23.9%	42.3%
HCV	72	36	0.69 (0.39-1.20)	0.43 (0.22-0.87)	68865 (56956-76210)	66.6%	82.6%
Uninfected	100	53	0.71 (0.47-1.08)	0.91 (0.52-1.60)	726249 (496338-926135)	2.7%	5.8%
**BCLC stage**							
A	8	6	NA	NA			
B	52	26	0.65 (0.33-1.30)	1.09 (0.33-3.53)	3632824 (2565687-5635283)	0%	0%

C	276	133	0.58 (0.45-0.75)	0.54 (0.39-0.75)	156832 (141891-175900)	31.6%	50.4%

Abbreviations: PFS, progression-free survival; OS, overall survival; QALY, quality-adjusted life-year; ICER, incremental cost-effectiveness ratio; WTP, willingness-to-pay; Atezo, atezolizumab; ECOG, Eastern Cooperative Group; MVI, macro-vascular invasion; AFP, alpha-fetoprotein; PD, disease progression; BCLC, Barcelona clinic liver cancer; HCV, Hepatitis C; HBV, Hepatitis B; HR, hazard ration; AFP, alpha fetoprotein.

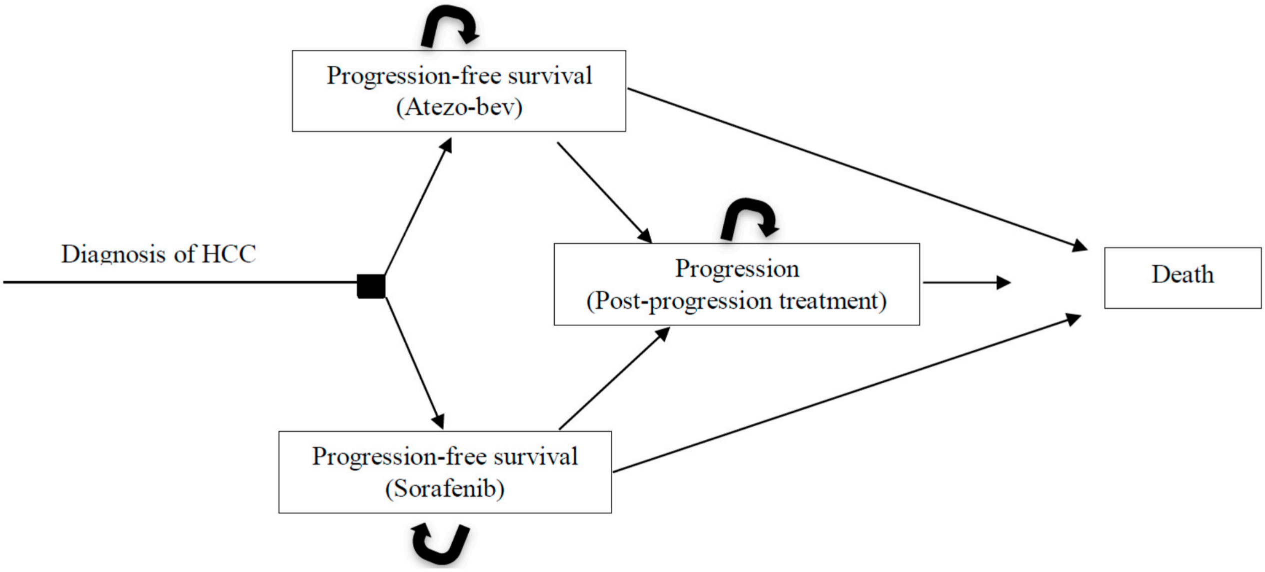

Figure S1. Simplified Markov model.

The three main health states are represented by ovals and include 'progression-free', 'disease progression', and "death." Arrows represent possible transitions from one health state to the next.

	Atezo-bev	Sorafenib
**OS ***		
Exponential	974	598
Weibull	959	591
Log-normal	961	593
Log-logistic	962	596
**PFS ***		
Exponential	1610	762
Weibull	1598	743
Log-normal	1609	748
Log-logistic	1604	753

*A lower AIC value indicates a better fit. Abbreviations: Atezo, atezolizumab; Bev, bevacizumab; OS, overall survival; PFS, progression-free survival.

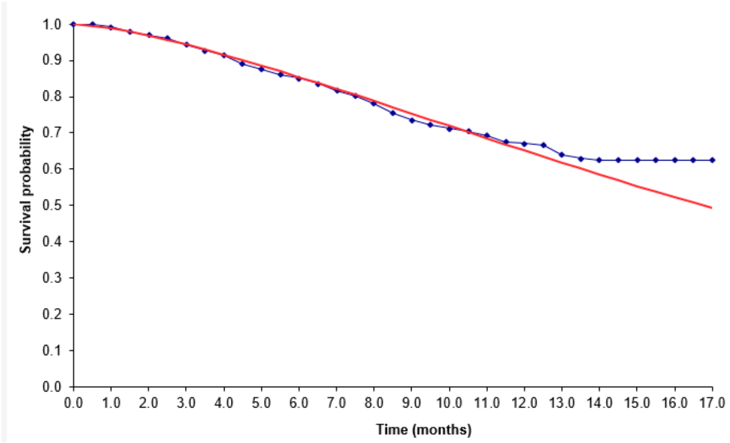
Survival probability
1.0
0.9
0.8
0.7
0.6
0.5
0.4
0.3
0.2
0.1
0.0
0.0 1.0 2.0 3.0 4.0 5.0 6.0 7.0 8.0 9.0 10.0 11.0 12.0 13.0 14.0 15.0 16.0 17.0
Time (months)

A

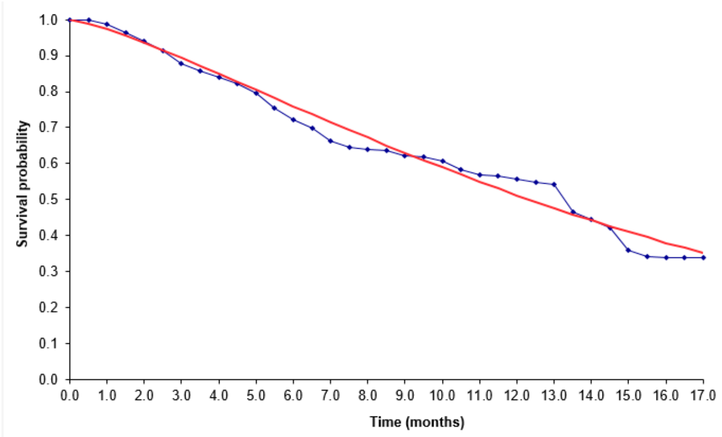
Survival probability
1.0
0.9
0.8
0.7
0.6
0.5
0.4
0.3
0.2
0.1
0.0
0.0 1.0 2.0 3.0 4.0 5.0 6.0 7.0 8.0 9.0 10.0 11.0 12.0 13.0 14.0 15.0 16.0 17.0
Time (months)

B

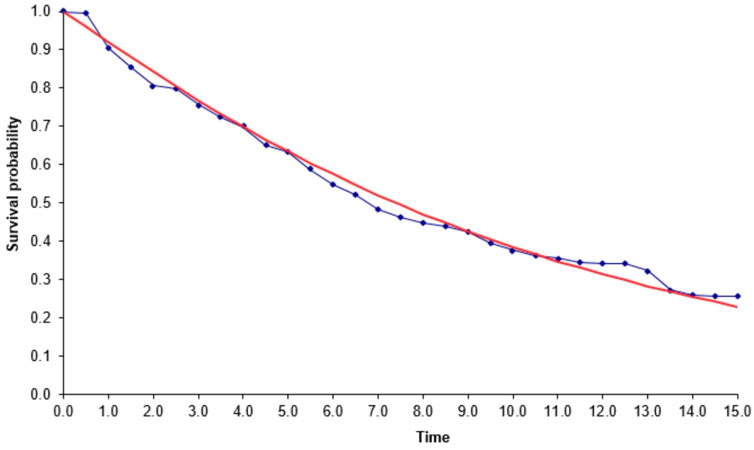
Survival probability
1.0
0.9
0.8
0.7
0.6
0.5
0.4
0.3
0.2
0.1
0.0
0.0 1.0 2.0 3.0 4.0 5.0 6.0 7.0 8.0 9.0 10.0 11.0 12.0 13.0 14.0 15.0
Time

C

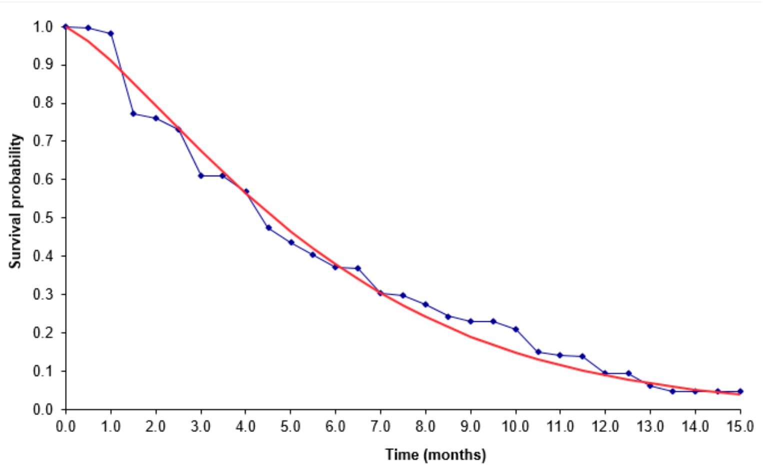

D

Figure S2. Quantitative measures of goodness of fit using AIC: Comparison of reconstructed survival curves. **A**. Weibull fit (red curve) of the observed overall survival curve (blue line) in atezo-bev arm; **B**. Weibull fit (red curve) of the observed overall survival curve (blue line) in sorafenib arm; **C**. Weibull fit (red curve) of the observed progression-free survival curve (blue line) in atezo-bev arm; **D**. Weibull fit (red curve) of the observed progression-free survival curve (blue line) in sorafenib arm

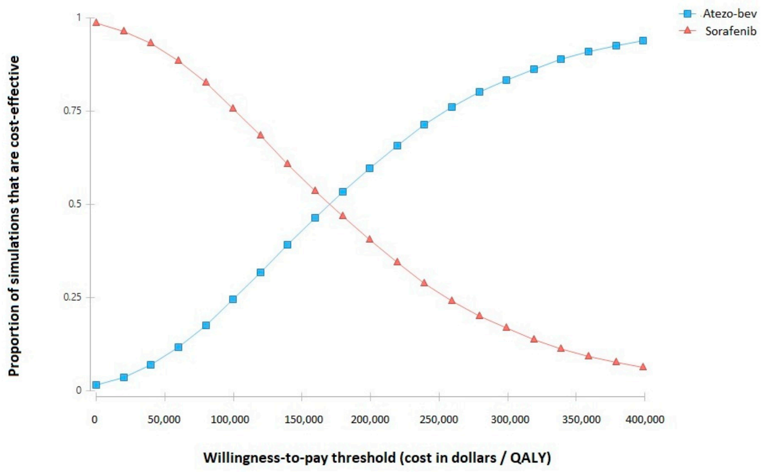

(A)

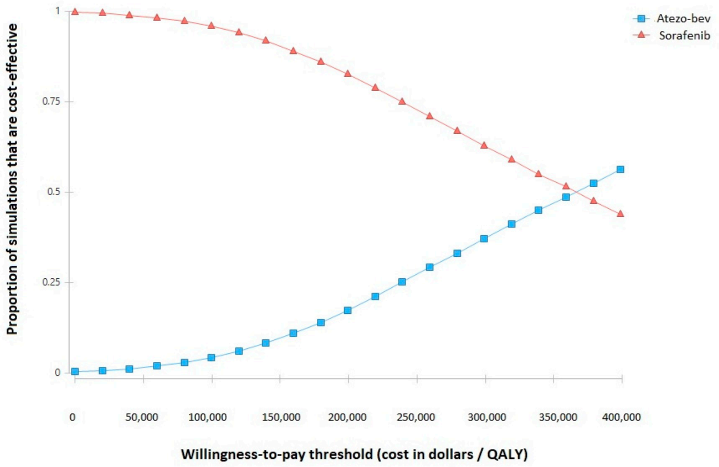

(B)

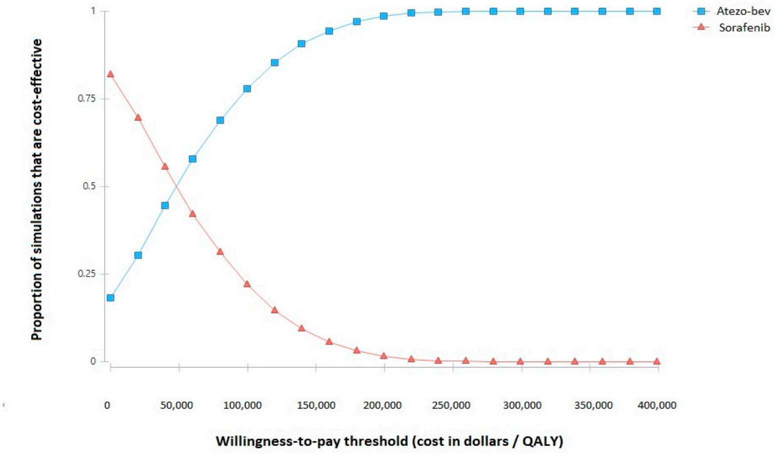

(C)

Figure S3. Cost-effectiveness acceptability curves: (**A**) base case *; (**B**) pessimistic survival estimation *; (**C**) optimistic survival estimation *.

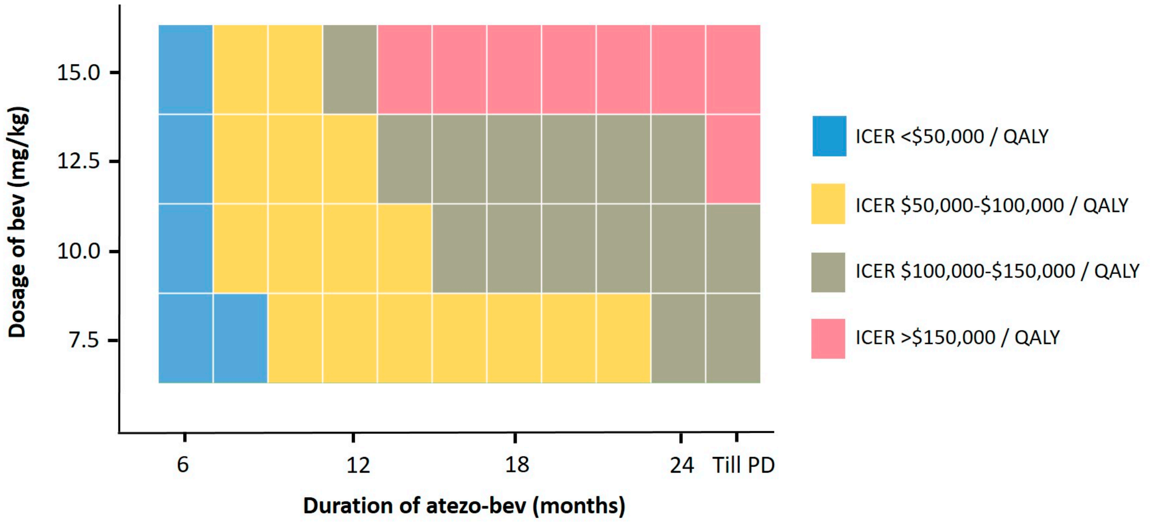

(A)

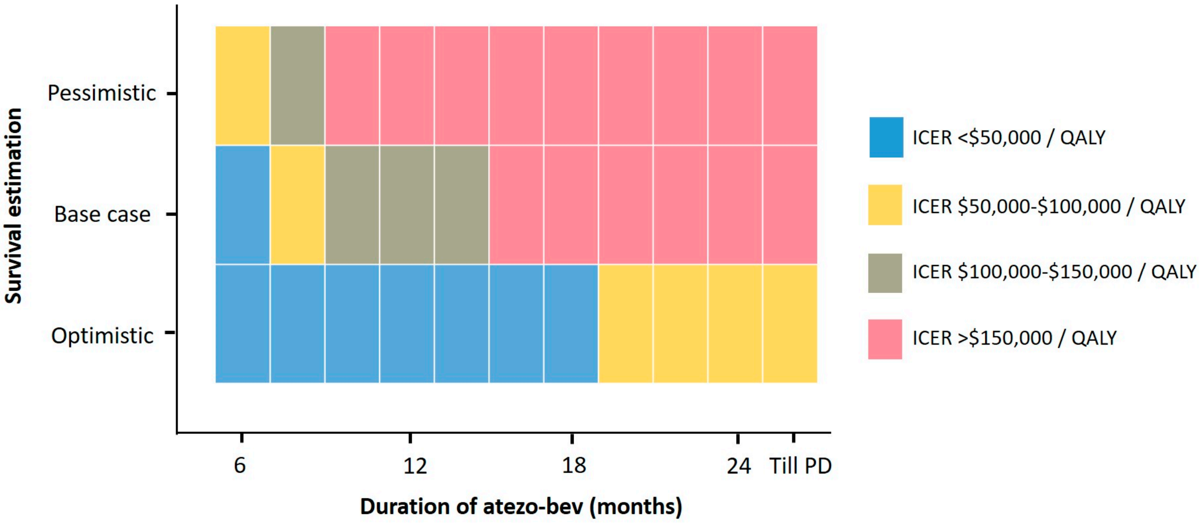

(B)

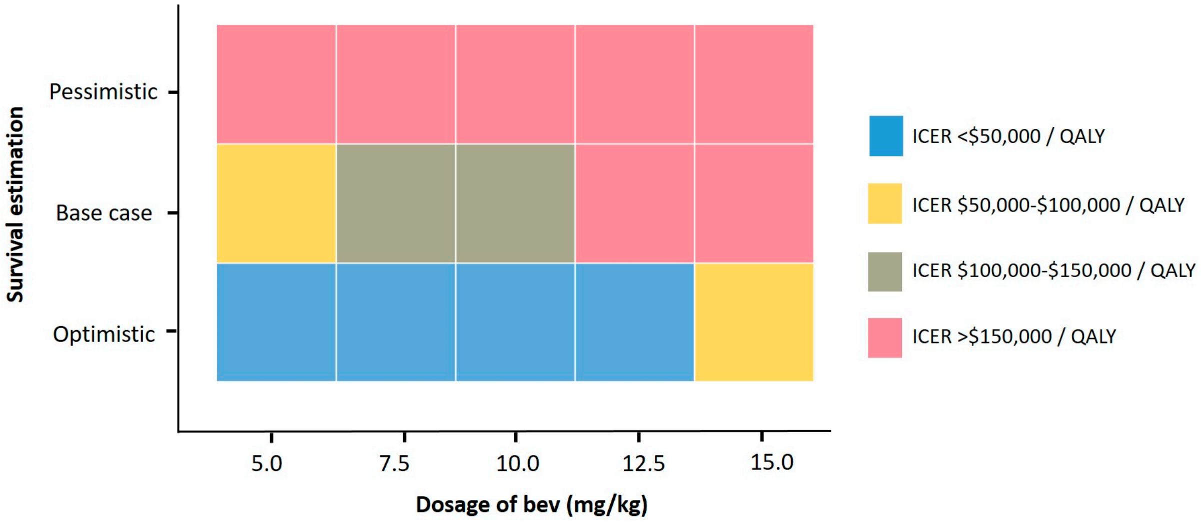
Pessimistic
Base case
Optimistic
Survival estimation
5.0
7.5
10.0
12.5
15.0
Dosage of bev (mg/kg)
ICER <$50,000 / QALY
ICER $50,000-$100,000 / QALY
ICER $100,000-$150,000 / QALY
ICER >$150,000 / QALY

(C)

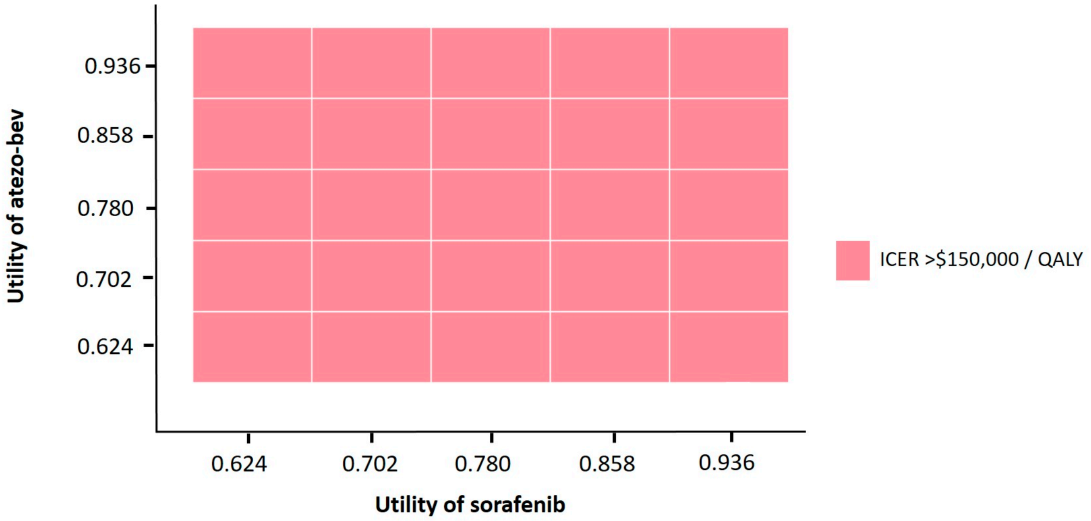
0.936
0.858
0.780
0.702
0.624
Utility of atezo-bev
0.624
0.702
0.780
0.858
0.936
Utility of sorafenib
ICER >$150,000 / QALY

(D)

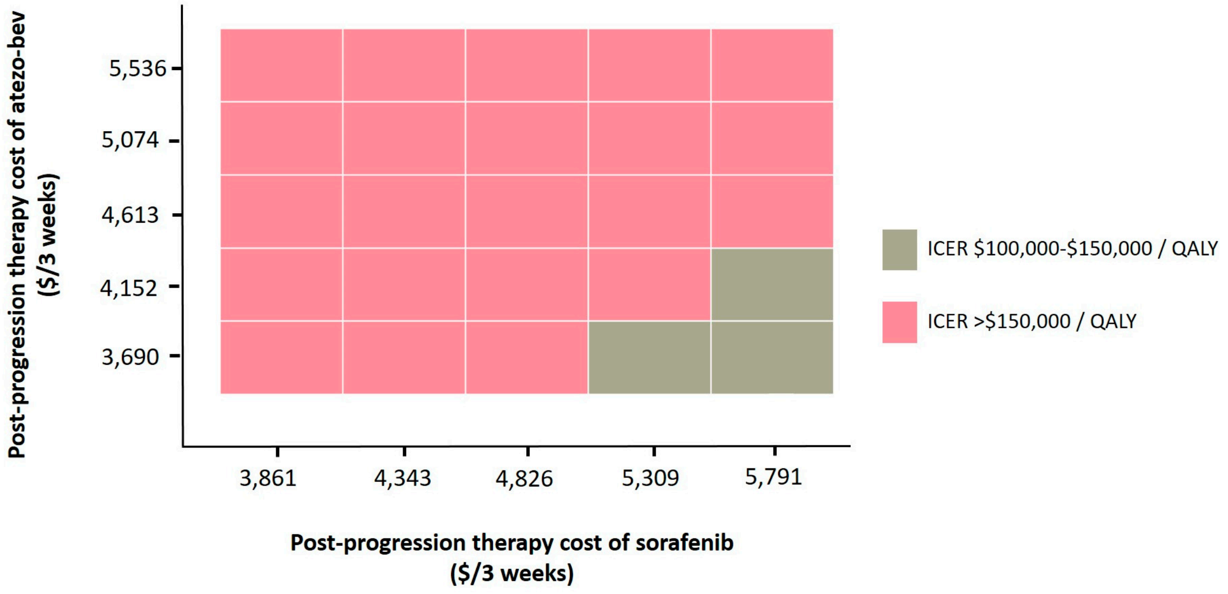

(E)

Figure 4. Results of two-way sensitivity analysis (**A**). Cost-effectiveness of Atezo-Bev combination compared with dosage and duration of Bev. The atezo-bev combination costs < $150,000 per QALY when the duration of atezo-bev is capped at 12 months. When the bev dosage is reduced to 10 mg/kg, atezo-bev is consistently cost-effective at frequently accepted thresholds. Abbreviations: Atezo, atezolizumab; Bev, bevacizumab; ICER, incremental cost-effectiveness ratio; QALY, quality-adjusted life-year; (**B**). Cost-effectiveness of Atezo-Bev combination compared with duration of Bev and survival estimation; The atezo-bev combination costs < $100,000 per QALY in the optimistic survival estimation; however, the atezo-bev combination costs > $150,000 in most scenarios under the pessimistic survival estimation. Abbreviations: Atezo, atezolizumab; Bev, bevacizumab; ICER, incremental cost-effectiveness ratio; QALY, quality-adjusted life-year. #Base case scenario: extrapolated long-term outcome from short-term data of IMbrave 150 study (i.e. 3-year survival rate of 37.7%); Pessimistic scenario: we assumed that survival after 17 months would follow the survival estimates of the US population with advanced HCC obtained from the SEER database (i.e. 3-year survival rate of 27.8%); Optimistic scenario: we assumed that all patients 'alive' at 17 months were 'cured' and the risk of death would be equal to their age-adjusted background mortality rate (i.e. 3-year survival rate of 60.7%); (**C**) Cost-effectiveness of Atezo-Bev combination compared with bev dosage and survival estimation. The atezo-bev cost < $100,000 per QALY in the optimistic survival estimation, whereas the atezo-bev combination costs > $150,000 in all scenarios under the pessimistic survival estimation. Abbreviations: Atezo, atezolizumab; Bev, bevacizumab; ICER, incremental cost-effectiveness ratio; QALY, quality-adjusted life-year. # Base case scenario: extrapolated long-term outcome from short-term data of IMbrave 150 study (i.e. 3-year survival rate of 37.7%); Pessimistic scenario: we assumed that survival after 17 months would follow the survival estimates of the US population with advanced HCC obtained from the SEER database (i.e. 3-year survival rate of 27.8%); Optimistic scenario: we assumed that all patients 'alive' at 17 months were 'cured' and the risk of death would be equal to their age-adjusted background mortality rate (i.e. 3-year survival rate of 60.7%); (**D**) Two-way sensitivity analyses for utility value of atezo-bev and sorafenib. (**E**) Two-way analyses for post-progression therapy costs of atezo-bev and sorafenib. Abbreviations: ICER, incremental cost-effectiveness ratio; Atezo, atezolizumab; Bev, bevacizumab; QALY, quality-adjusted life-year; Abbreviations: ICER, incremental cost-effectiveness ratio; Atezo, atezolizumab; Bev, bevacizumab; QALY, quality-adjusted life-year.
